# Regulatory chromatin landscape in *Arabidopsis thaliana* roots uncovered by coupling INTACT and ATAC-seq

**DOI:** 10.1186/s13007-018-0381-9

**Published:** 2018-12-20

**Authors:** Miriam Tannenbaum, Avital Sarusi-Portuguez, Ronen Krispil, Michal Schwartz, Olga Loza, Jennifer I. C. Benichou, Assaf Mosquna, Ofir Hakim

**Affiliations:** 1 The Mina and Everard Goodman Faculty of Life Sciences, Bar-Ilan University, Ramat-Gan, Israel; 2 Robert H. Smith Institute of Plant Sciences and Genetics in Agriculture, Faculty of Agriculture, Hebrew University of Jerusalem, Rehovot, Israel

**Keywords:** Chromatin accessibility, Chromatin structure, Regulatory element, Transcription, Transcription factor, *Arabidopsis thaliana*, ATAC-seq, INTACT

## Abstract

**Background:**

There is a growing interest in the role of chromatin in acquiring and maintaining cell identity. Despite the ever-growing availability of genome-wide gene expression data, understanding how transcription programs are established and regulated to define cell identity remains a puzzle. An important mechanism of gene regulation is the binding of transcription factors (TFs) to specific DNA sequence motifs across the genome. However, these sequences are hindered by the packaging of DNA to chromatin. Thus, the accessibility of these loci for TF binding is highly regulated and determines where and when TFs bind. We present a workflow for measuring chromatin accessibility in *Arabidopsis thaliana* and define organ-specific regulatory sites and binding motifs of TFs at these sites.

**Results:**

We coupled the recently described isolation of nuclei tagged in specific cell types (INTACT) and assay for transposase-accessible chromatin with high-throughput sequencing (ATAC-seq) as a genome-wide strategy to uncover accessible regulatory sites in *Arabidopsis* based on their accessibility to nuclease digestion. By applying this pipeline in *Arabidopsis* roots, we revealed 41,419 accessible sites, of which approximately half are found in gene promoters and contain the H3K4me3 active histone mark. The root-unique accessible sites from this group are enriched for root processes. Interestingly, most of the root-unique accessible sites are found in nongenic regions but are correlated with root-specific expression of distant genes. Importantly, these gene-distant sites are enriched for binding motifs of TFs important for root development as well as motifs for TFs that may play a role as novel transcriptional regulators in roots, suggesting that these accessible loci are functional novel gene-distant regulatory elements.

**Conclusions:**

By coupling INTACT with ATAC-seq methods, we present a feasible pipeline to profile accessible chromatin in plants. We also introduce a rapid measure of the experiment quality. We find that chromatin accessibility at promoter regions is strongly associated with transcription and active histone marks. However, root-specific chromatin accessibility is primarily found at intergenic regions, suggesting their predominance in defining organ identity possibly via long-range chromatin interactions. This workflow can be rapidly applied to study the regulatory landscape in other cell types, plant species and conditions.

**Electronic supplementary material:**

The online version of this article (10.1186/s13007-018-0381-9) contains supplementary material, which is available to authorized users.

## Background

As sessile organisms, plants can accommodate to changes in the environment by modulating their cellular transcription programs to allow developmental plasticity [1], which requires plant-specific transcriptional regulatory mechanisms that defines these programs. Despite the ever-growing genome-wide gene expression data, understanding how transcriptional programs are established and regulated to define cell identity is largely lacking.

The genome regulatory potential relies not only on DNA sequence but also on epigenetic features that dictate the time, place and level of gene transcription. The chromatin structure is highly regulated and dynamic as histones can be evicted from chromatin to expose regulatory sites and allow binding of transcription factors (TFs) and other regulatory proteins to DNA [2] or be recruited back to hinder the regulatory DNA. Thus, the cell type-specific profile of chromatin accessibility to TF binding is an essential layer of gene regulation. The regulatory circuitry that determines the cellular transcription program relies on a network of multiple TFs and their association with cell-type-selective accessible regulatory sites [3]. Genome-wide mapping of TF binding to chromatin is commonly performed by chromatin immunoprecipitation (ChIP)-based methods, such as ChIP-chip and ChIP-seq. However, these techniques rely on prior knowledge of candidate factors and the availability of a specific antibody or generation of a transgenic line expressing a tag fused to a target protein. Given the lack of specific antibodies or tagged target proteins for more than one thousand TFs in plants (for example, over 1900 TFs were identified in *Arabidopsis* [4]), ChIP-based approaches are difficult to apply on a large scale. Thus, novel unbiased genomic approaches are needed to uncover the target loci of these factors, which are largely unknown.

TFs can bind specific DNA sequence motifs. Thus, uncovering these motifs in active *cis*-regulatory elements (CREs) is a powerful approach for predicting their associated TFs and depicting the gene regulatory network underlying a transcriptome [2, 5]. The applicability of this approach has dramatically increased with the recent introduction of the DNA affinity purification sequencing (DAP-seq) method that uncovered 219 novel TF binding motifs in *Arabidopsis* [5].

**Fig. 1:**
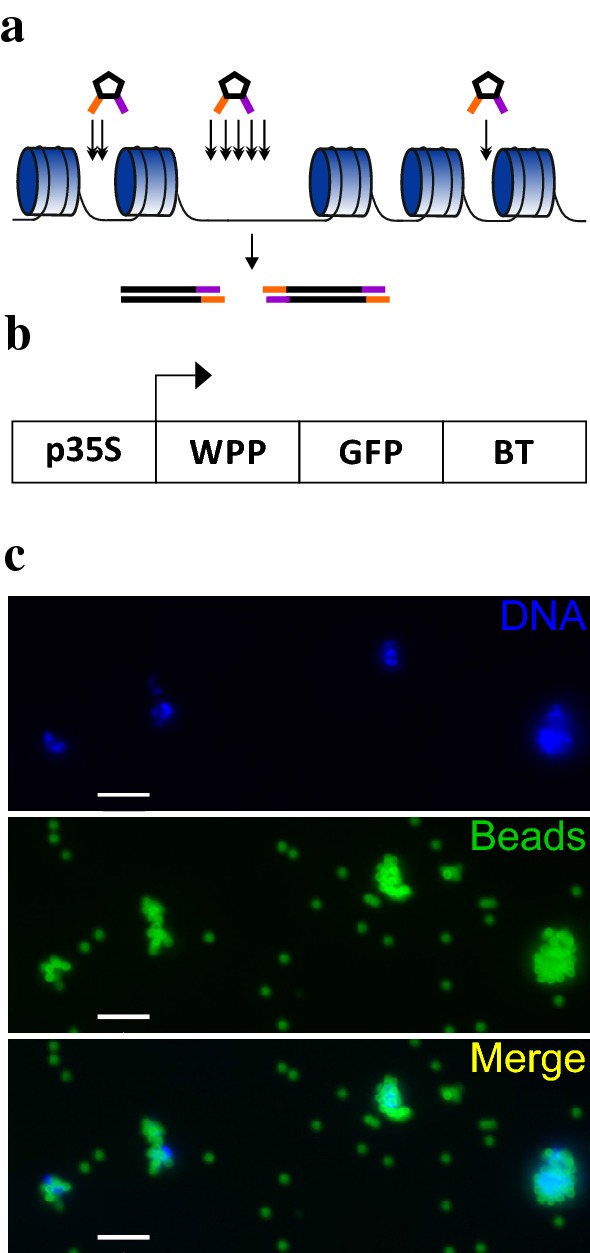
Overview of methods. **a** Scheme depicting the ATAC-seq method. Transposase (pentagons) digests the chromatin more often in accessible sites and deposits its adapters (orange and purple), which are used for library preparation and sequencing. **b** Illustration of the INTACT construct. p35S, 35S promoter; WPP, nuclear envelope targeting domain; GFP, green fluorescent protein; BT, biotinylatable tag. **c** Nuclei isolated using the INTACT method. The nuclei are stained with DAPI (blue) and are bound by streptavidin-coated beads (green). Scale bar: 10 µm

Almost all active CREs, including promoters, enhancers, suppressors, and insulators, are located at chromatin accessible sites, which are hypersensitive to cleavage by endonucleases, such as DNaseI or transposase [6]. In many studies, accessible sites were identified by DNaseI digestion followed by blotting or sequencing [7–10]. Although the DNaseI method has existed for over 30 years, global DNaseI hypersensitive site (DHS) profiling in plants has only recently become feasible [4, 6–8, 11]. However, the large number of pure intact nuclei required for the DNaseI method is a limiting factor for its application. A recent ATAC-seq method (Assay for Transposase-Accessible Chromatin with high-throughput sequencing) [12] (Fig. 1a) exhibits great promise for overcoming this limitation given that it requires much less starting materiel. In ATAC-seq, chromatin cleavage is performed by Tn5 transposase, which introduces a DNA adaptor to the cleaved site. In addition to the smaller number of nuclei needed compared with DHS-seq, the ATAC-seq protocol is faster and requires fewer steps, therefore introducing lower bias [13]. Since its publication approximately four years ago, ATAC-seq has been applied in various studies, including basic biology [14–16] and disease research [14, 17–20]. In addition, ATAC-seq has recently been applied in rice [21] and *Arabidopsis* [22]. The INTACT method allows isolation of nuclei from individual cell types by affinity purification based on expression of a biotinylated nuclear envelope protein [23, 24] (Fig. 1). Merging the two approaches in *Arabidopsis thaliana* allows depicting of organ or cell type-specific chromatin accessibility landscape [15, 16, 25].

Here, we report the combination of these two powerful methods to measure the chromatin accessibility landscape in *Arabidopsis thaliana* roots. We demonstrated that chromatin accessibility at gene promoters is associated with gene expression and H3K4me3 histone marks in *Arabidopsis* roots. Interestingly, the majority of root-unique accessible regulatory loci are nongenic and correlated with root-specific expression of distant genes. This finding strongly suggests that organ specificity is predominantly associated with variation in gene regulation rather than expression and that chromosome looping may be an important layer of gene regulation in plants.

## Methods

### Plants material and growth conditions

*Arabidopsis thaliana* Columbia ecotype was grown in growth chambers under cool white LED light ~ 100 LUX for 16-h light/8-h dark at 22 °C. For root collection, seeds were surface sterilized with chlorine steam using 100 ml bleach (v/v, 5% sodium hypochlorite) and 6 ml HCl (v/v, 32% hydrochloric acid). Seeds were sowed on square Petri dishes coated with 1/8 strength MS (Murashige and Skoog) media pH 5.7 [26] and incubated vertically. Roots were harvested 14 days after sowing.

### Cloning the INTACT plasmid

To generate plants expressing the INTACT [23, 24] system under the constitutive 35S promoter, the 5′ end of the AT3G63130 gene encoding amino acids 1–111 of RanGAP1 (WPP-domain) was amplified using primers bearing AgeI and XmaI at the 5′ and 3′ ends, respectively (5′- AAAAAAACCGGTATGGATCATTCAGCGAAAACCACAC-3′ and 5′- AAAAAACCCGGGAGCGGCCGCCTCAACCTCGGATTCTTCCTGTG-3′). Following restriction, the latter was ligated to pEGHPB [27, 28] at the AgeI site. The construction of pEGHPB was described in [27]. In short, HA tag–PreScission–Biotin (HPB) was chemically synthesized and cloned into pEGAD [28, 29]. The biotin target sequence is the C-terminal of Bioing Carboxyl Carrier Protein domain (BCCD) of *Arabidopsis* 3-methylcrotonal CoA carboxylase [29]. This system resulted in a 35S-driven WPP domain for nuclear envelope targeting fused to GFP and HPB. This system negates the need for coexpression of bacterial BirA biotin ligase given that BCCD is biotinylated in vivo. *Arabidopsis thaliana* (Col-0) was stably transformed with this construct by floral dip [30]. Transformants were selected based on GFP signal, and several T_3_ homozygous, single copy, independent transformants were acquired.

### Nuclei purification

For purifying 100,000–150,000 nuclei, 1 g of fresh tissue was harvested and ground in liquid nitrogen using a mortar and pestle to break the cell wall. To avoid enzymatic activity, all the following steps were performed at 4 °C. The resulting powder was transferred into 20 mL NPB (Nuclear purification Buffer containing 20 mM MOPS, 40 mM NaCl, 90 mM KCl, 2 mM EDTA pH 8, 0.5 mM EGTA, 0.2 mM Spermine, 0.5 mM Spermidine and protease inhibitor × 1 (Sigma P2714)) and incubated on a rotator at 4 °C for 10 min. The sample was then transferred through a 40-µm mesh and centrifuged for 10 min at 1000 g and 4 °C. The pellet was gently resuspended in 7 mL NPBt (NPB supplemented with 0.1% Triton X-100). Nuclei were then bound to 25 µl of streptavidin-coated Dynabeads (Invitrogen, M-280 Strepavidin) according to the manufacturer’s protocol and washed with NPB. After washing 4–5 times with 1 mL NPBt, the nuclei were resuspended in 1 mL NPB. To count nuclei, a 20-µl suspension was loaded to a Marienfeld hemocytometer (Neubauer-improved, chamber depth of 0.1 mm, 0630010), and visualized with a Nikon Eclipse TS100 light microscope.

### ATAC-seq

To remove EDTA, nuclei were washed with a low volume of TDX buffer (Illumina, cat. No. FC-121-1030). Transposition was performed as published [12, 13] with several modifications. Briefly, purified nuclei were resuspended in 22.5 µl TDX and transposed using 2.5 µl of the Tn5 enzyme from the Nextra kit (Illumina, cat. No. FC-121-1030) for 15 min at 37 °C in a thermal shaker. DNA was then purified by PCR Extraction Kit (hy-labs, EX-GP200), and PCR reactions for library amplification were performed as described previously [12, 13]. Briefly, the libraries were amplified for five cycles followed by qPCR on 10% of the reaction to assess the number of additional cycles needed (6–9 additional PCR cycles). The libraries were size selected by a gel-free double-sided size-selection protocol using Agencourt AMPure XP beads (Cat. No. 63881) at 0.5X and 1.2X. DNA was quantified by Qubit HS DNA kit (Thermo Fisher, Q32854), and analyzed on a BioAnalyzer or TapeStation. Libraries were sequenced on an Illumina HiSeq 2500 sequencer, and 61 bp were sequenced using a single-end protocol.

### Quality control for ATAC-seq libraries by qPCR

The enrichment of accessible regions relative to inaccessible regions was measured by qPCR using DNA from the final ATAC-seq libraries. qPCR was performed using the iTaq universal SYBR green supermix (Bio-Rad, 172-5124). The final volume of each reaction was 10 μl. Primers were designed using the NCBI primer-BLAST. The final concentration of the primers was 0.5 μM. All amplicons were 90 bp long. Each PCR plate contained genomic DNA, and normalization was performed using the following equation: (primer efficiency)^(average cq gDNA − average cq sample)^ for each primer set. To define the fold change for each library, the average of normalized amplification of all accessible (open) regions was divided by the average of normalized amplification of all inaccessible (closed) regions. qPCR thermal cycles were as follows: 3 min 95 °C; 39X (10 s 95 °C, 30 s 60 °C).

The following qPCR primers were used to amplify accessible loci (5′–3′): Set 1: forward: TGGAATACACCAGAGAAAGGATAAC, reverse: GCATAAGTGATTTCATTCTGCGA. Set 2: forward: AGCCCATTCAAGGCCTCACA, reverse: GGCAATTCGAAGTTGAAGGCAT.

The following qPCR primers were used to amplify inaccessible loci (5′–3′): Set 3: forward: ATCATATTCTTCACAGTTTGATCCC, reverse: ACATTTCAGGTTGGGAGACAGA. Set 4: forward: CCCAGGGGAATACGGTCAAC, reverse: AAGAGCTTACGAAACTGGAGGT.

### RNA-seq

RNA was isolated from 14-day-old roots using TRIZOL (Life Technologies) reagent and cleaned with RNA Clean & Concentrator™-5 (Zymo, R1015). RNA was quantified by NanoDrop2000 (Thermo Fisher). Libraries were prepared with the NEBNext RNA library prep kit (module E7530-E7490). cDNA was quantified using a Qubit HS DNA kit (Thermo Fisher, Q32854) and analyzed on a BioAnalyzer or TapeStation. Libraries were sequenced on an Illumina HiSeq 2500 sequencer, and 61 bp were sequenced using a single-end protocol.

### Data analysis

Sequencing reads were aligned to the TAIR10 *Arabidopsis* genome using bowtie [31] with the −m 1 option, retaining reads that map to a sole location. PCR duplicates were removed using the SAMtools [32] rmdup option. Peaks were called by applying MACS2 [33] with the following parameters: -g 135000000–nomodel–extsize 75–shift-30. Reproducibility was calculated using the lm() function in the R programming language. Motifs were identified using HOMER [34]. Leaf DHS-seq data (GSE34318) were used from [7]. Leaf RNA-seq data (GSE38612) were downloaded from the GEO database. Reads were aligned to the TAIR10 *Arabidopsis* genome using TopHat2. To calculate the number of reads on each gene, HT-seq was used, and RPKM was calculated using EdgeR package in the R programming language. GO analysis was performed using the GO Ontology database (release 2017-06-29). Sequencing data of histone modifications ChIP-seq were downloaded (E-MTAB-1663). Reads were aligned to the TAIR10 *Arabidopsis* genome with bowtie. Only unique mapped reads were used. Peaks were called by applying MACS2 with the following parameters: -g 135000000–nomodel–extsize 143. Analyzed PLT2 ChIP-seq data (GSE79755) were downloaded from [35], and the ChIP-seq peak summits were intersected with the ATAC-seq peaks. Then, Chi squared tests of independence were performed. Odds ratios (ORs) between proportions of accessible sites in leaf and root in both unique and all sites were estimated, and their differences were tested for significance. Specifically, a Z-score was calculated by taking the difference of the log ORs and dividing it by their pooled standard error. Finally, a two-tailed *p* value was obtained from the normal standard distribution. To calculate the expression of genes near intergenic accessible sites, the nearest gene was identified using annotatePeaks in the HOMER [34] package, and the RPKM values were obtained (each gene was represented only once). Student’s t-test was calculated between the RPKM of the genes in roots and leaves. To calculate the significance of the magnitude, a log_2_ value was calculated for each gene in the group of genes near intergenic root unique accessible sites and in the group of genes near intergenic root nonunique accessible sites. Finally, Student’s t-test was performed.

## Results

### Isolation of nuclei

In this study, we combined novel methodologies for rapid and efficient measuring global chromatin accessibility in *Arabidopsis thaliana* and uncovering active regulatory elements that are key for regulating tissue-specific transcriptional programs. Although chromatin accessibility can be profiled by other methodologies, such as DNase-seq, these techniques are not widely used due to technical limitations. The integrity of isolated nuclei is critical for profiling chromatin accessibility given that their disruption by mechanical shearing reduces the signal-to-noise ratio [13]. Using the INTACT method (Fig. 1b, c), we obtained intact nuclei, which is important for preserving chromatin integrity. In addition, the high purity of the isolated nuclei is important for reducing nonnuclear DNA contamination in the downstream DNA sequencing reaction. The proportion of contamination by mitochondrial DNA in ATAC-seq libraries from animal cells ranges from 30 to 70% [13, 22]. Nonnuclear DNA contamination is expected to be increased in plants, which also contain chloroplasts. Eliminating the nonquantifiable and variable chloroplast and mitochondria DNA contamination is also important for calculating the proper ratio of Tn5 transposase and nuclear DNA for the tagmentation reaction. Altogether, we were able to minimize DNA contamination levels to ~ 2% as described below.

### Preparation of the ATAC-seq libraries

Plant cells have a vacuole, which contains various hydrolytic enzymes, including DNase. Unlike lysosomes in animal cells, which remain intact and are discarded during nucleus isolation, in plants, the vacuole membrane is disrupted and its contents are released, leading to undesirable DNA cleavage. Affinity purification of nuclei by INTACT allows the cellular extract to be rapidly and efficiently discarded. Moreover, to minimize nonspecific DNA degradation due to the nuclease-promoting conditions, we also reduced the tagmentation reaction time (see “Methods” section).

Preferential cleavage of accessible sites relative to inaccessible chromatin relies on the ratio between transposase enzyme and nuclei. Excess enzyme would lead to excessively frequent cleavage; thus, enrichment for DNA from accessible sites would be lost. On the other hand, enzyme insufficiency reduces the detection sensitivity [13]. Hence, quantifying the nuclei is critical for calibrating the nuclear-enzyme ratio. According to recent studies, this ratio is organism specific [14, 36] and thus should be calibrated empirically.

As shown in Fig. 1c, affinity purified nuclei tend to cluster with each other on strepavidin beads. For consistency and simplicity, we calculated the ratio between nuclear clusters and the Tn5 enzyme. To calibrate the enzyme-nuclei ratio, we used a constant amount of Tn5 enzyme with a range (~ 25,000–100,000) of nuclei. Similar to reports for human cells [13], we note that a variation in cell numbers of ± 50% does not have a major effect on the quality of the results and thus conclude that the approach is robust in *Arabidopsis*, and within this nuclei range.

### Quality of ATAC-seq libraries assessed prior to sequencing

**Fig. 2:**
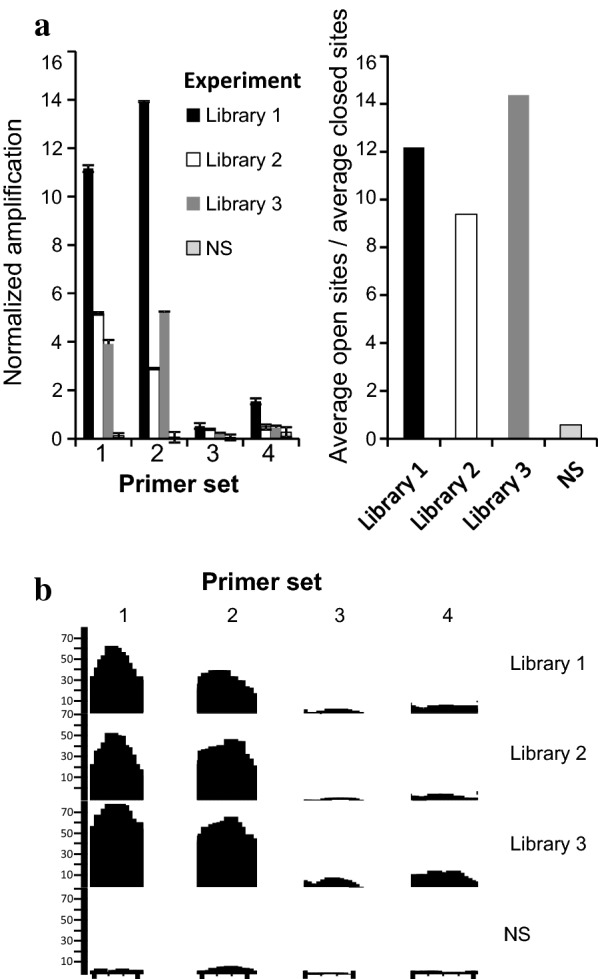
Quality control of ATAC-seq libraries. **a** Chromatin accessibility at four genomic loci (90 bp) measured by qPCR. Left, the amplification of each region was normalized to genomic DNA at the same locus. Primer sets 1 and 2 amplify open (accessible) regions, whereas primer sets 3 and 4 amplify closed (inaccessible) regions. Right, quality control score calculated as the ratio of the average of the two open versus closed sites. NS—an example of a library with nonspecific digestion. Error bars indicate standard deviation of three technical repeats. ***p < 0.001, ANOVA and Tukey’s test. For NS library, p = 0.95 **b** Genomic browser view of the regions amplified in (**a**) after sequencing (aligned to TAIR10)

Despite its relative simplicity, calibrating and performing ATAC-seq remain challenging. Therefore, we developed a quality control step that enables us to assess the enrichment of accessible chromatin relative to the inaccessible genomic background in the ATAC-seq DNA sample. Its application prior to next-generation sequencing (NGS) saves time and money. To develop such a control that could also be applied in *Arabidopsis* organs in which the chromatin accessibility profile is unknown, we designed a qPCR-based approach to define ATAC-seq library quality [37] by assessing the enrichment of accessible loci versus inaccessible loci (Fig. 2). We searched for loci that exhibit indications of accessibility (or inaccessibility) by DNaseI hypersensitivity assay and indirectly by histone modifications using ChIP assay in multiple *Arabidopsis* cell types and organs [7, 9, 10, 38] (Additional file 1: Fig. S1). DNA fragments of   90 bp from these regions corresponding to the 100–800 bp DNA fragments in the ATAC-seq library following size selection [13] were amplified by qPCR. The quality of the ATAC-seq library was assessed by calculating the ratio between the enrichment of DNA from accessible chromatin loci relative to inaccessible chromatin loci (Fig. 2). Notably, given that the level of chromatin accessibility is locus specific, the particular enrichment level is related to the loci that were tested. We found that in cases in which this ratio was greater than 9, accessible chromatin loci were detected by NGS (Fig. 2). Given the difficulty of accurately quantifying the nuclei, we recommend performing the ATAC-seq experiment for a range of nucleus quantities and using this simple qPCR assay for selecting the best experiment for sequencing.

### The regulatory landscape of *Arabidopsis thaliana*

**Fig. 3:**
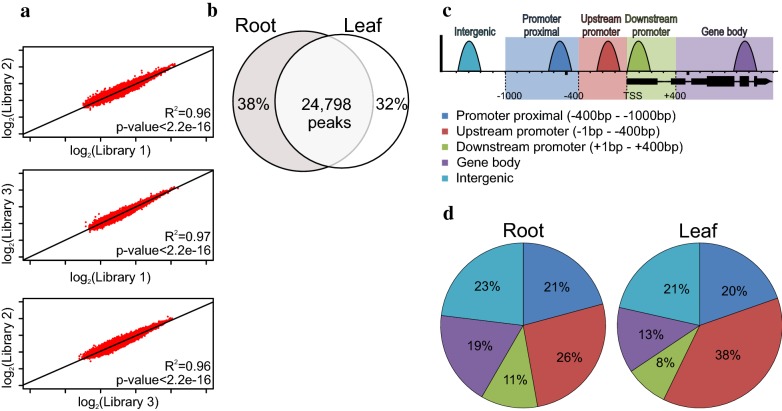
Quality of ATAC-seq libraries. **a** Peak intensity plots of ATAC-seq libraries from three biological Libraries (Pearson’s correlation). **b** Overlap between our root ATAC-seq data and published leaf DNase-seq data. **c** Schematic illustration of the genic position of peaks presented in **d**. **d** Genic distribution of accessible sites in root (left) and leaf (right)

After performing ATAC-seq for *Arabidopsis* roots, sequenced reads were mapped to the *Arabidopsis* genome (TAIR10), revealing that it contains only 2.15–1.43% nonnuclear DNA contamination (Additional file 1: Table S1). This finding indicates the high purity of the nuclei that was achieved by applying the INTACT isolation method. The ATAC-seq libraries generated from 25,000 to 100,000 nuclei were highly reproducible (Fig. 3a) and combined into a single dataset for further analysis. Uniquely mapped reads covered approximately 6.62% of the *Arabidopsis* genome, corresponding to 41,419 accessible sites in roots. The accessible loci measured in roots by ATAC-seq highly overlap those from leaf measured by DNase-seq and exhibit a similar distribution relative to genes [7], indicating their validity (Fig. 3b–d).

### High chromatin accessibility of active gene promoters correlates with transcription and histone modifications

**Fig. 4:**
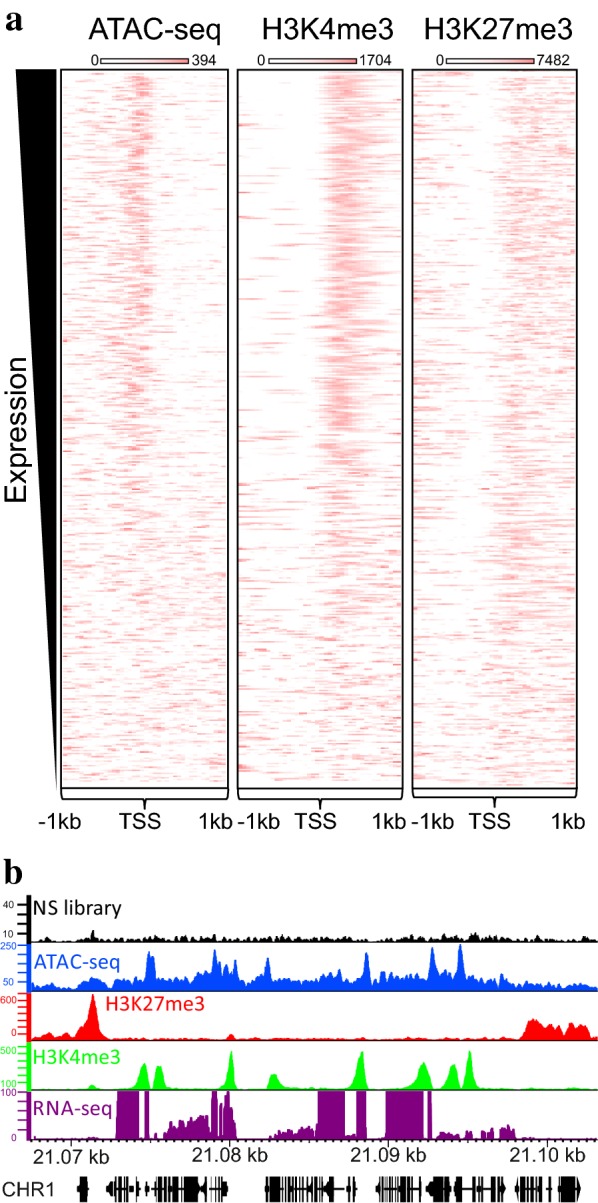
High chromatin accessibility at active genes correlates with histone modifications. **a** Heatmaps of ATAC-seq, ChIP-seq signal of active (H3K4me3) and repressive (H3K27me3) chromatin marks flanking (± 1 kb) TSS of genes ranked according to their expression level. **b** Browser view of a representative genomic locus (chromosome 1: 21,067,500–21,103,000). Black: ATAC-seq library with no significant peaks (see Fig. 2, NS). Blue: chromatin accessibility depicted by ATAC-seq. Red: H3K27me3 ChIP-seq profile. Green: H3K4me3 ChIP-seq profile. Purple: RNA-seq

To understand the link between accessible sites and gene expression, we ranked all of the *Arabidopsis* genes according to their RNA level from RNA-seq and found that highly expressed genes are accessible upstream of their transcription start site (TSS) (Fig. 4). This high accessibility is associated with the H3K4me3 active histone mark and does not correlate with the H3K27me3 repressive histone mark at these genes (Fig. 4). This concordance between chromatin accessibility and active histone mark is lost in genes that are expressed at a low level.

### Root-unique accessible sites at gene promoters correspond with organ-specific gene expression

To better understand the link between organ-specific chromatin accessibility and gene expression, we analyzed the root-specific (compared to leaves) accessibility profile and retrieved 989 genes with root-unique accessible site at their promoter region (− 1000 bp to + 400 bp). Approximately half (48.13%) of these genes are significantly upregulated in roots (vs. leaves, log_2_FC > 1, p-value < 0.05, RPKM > 1) compared with 5.76% of genes that are significantly upregulated in leaves from this group. We performed GO enrichment analysis on these 476 highly expressed genes. As expected, these genes were enriched with terms associated with root functions, such as response to salt stress (p-value < 9.29e−05) and response to osmotic stress (p-value < 3.92e−05) (Additional file 1: Fig. S2). This finding indicates that the organ-specific regulatory landscape reflects organ-specific gene expression.

### Accessible sites contain motifs and binding sites of root TFs

Selective activation of regulatory DNA elements defines sites at which TFs may bind and act. Thus, to predict the identity of TFs that are active in roots, we computed the enrichment of sequence motifs in the all of the root ATAC-seq accessible sites using HOMER (Additional file 1: Table S2). In total, 27 of the 30 highest-ranked motifs contain an E-box binding motif known as the G-box motif [39]. This motif, which was also found in DNase-seq from leaves [9], is a highly conserved DNA sequence that is required for the regulation of numerous plant genes [40]. This finding demonstrates the ability to identify biologically relevant motifs within ATAC-seq peaks.

**Fig. 5:**
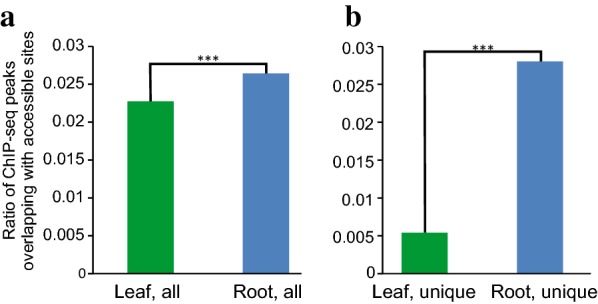
Accessible sites are associated with PLT2 binding. The proportion of accessible sites from leaves (green) or root (blue) associated with PLT2 binding from root ChIP-seq summits (1 bp). **a** All accessible sites, **b** tissue-specific accessible sites. ***p-value < 0.001 (*Chi* square test of independence). Odds ratio (OR) between leaf and root in unique sites was significantly larger than in all accessible sites (OR = 5.312498 and 1.167073 respectively, p < 0.001)

Similar to animals, binding of TFs to accessible chromatin in plants is reflected by overlapping ChIP-seq and chromatin accessibility peaks in an organ-specific manner [3, 9, 24, 41, 42]. To assess the organ-specific binding of root TF to root accessible chromatin, we assessed the overlap between the binding of the Plethora 2 (PLT2) transcription factor [35], which defines the quiescent center stem cell niche in *Arabidopsis* root [43], and chromatin accessibility (Fig. 5a). Given that many transcription factors, such as PLT2 are also expressed in shoot cells [43], we used PLT2 ChIP-seq from *Arabidopsis* roots. Importantly, PLT2 binding loci overlap root ATAC-seq to a higher degree than leaf accessible chromatin (p-value < 2.2e−16). Notably, this divergence was fivefold greater in the root-unique compared with the leaf-unique accessible sites (Fig. 5b) (p-value < 5.351e−16).

These results suggest that focused analysis of motif enrichment in the group of root-unique accessible sites will increase the capacity to discover root-specific transcription factors. Therefore, we searched for enriched motifs in the root-unique accessible sites, using the remainder of the root accessible sites as background. One of the top ranking motifs is the binding sequence of MYB61 transcription factor (rank 2, p-value < 1e−8), which is also expressed uniquely in roots (tenfold increase in roots relative to leaves). MYB61 regulates root growth and architecture by controlling multiple aspects of plant resource allocation [44, 45]. The fact that MYB61 ranked only 130 (p-value < 1e−48) in the group of motifs from all root accessible sites demonstrates the capability of revealing root-specific factors from the focused list (Additional file 1: Table S3). Hence, we conclude that the ATAC-seq profile from roots retrieved cis-accessible regions that contain binding sites for root-specific transcription factors and thereby represent bona fide regulatory elements important for regulating root-specific transcriptional program.

### Organ-specific variance in chromatin accessibility is predominantly associated with gene regulation

**Fig. 6:**
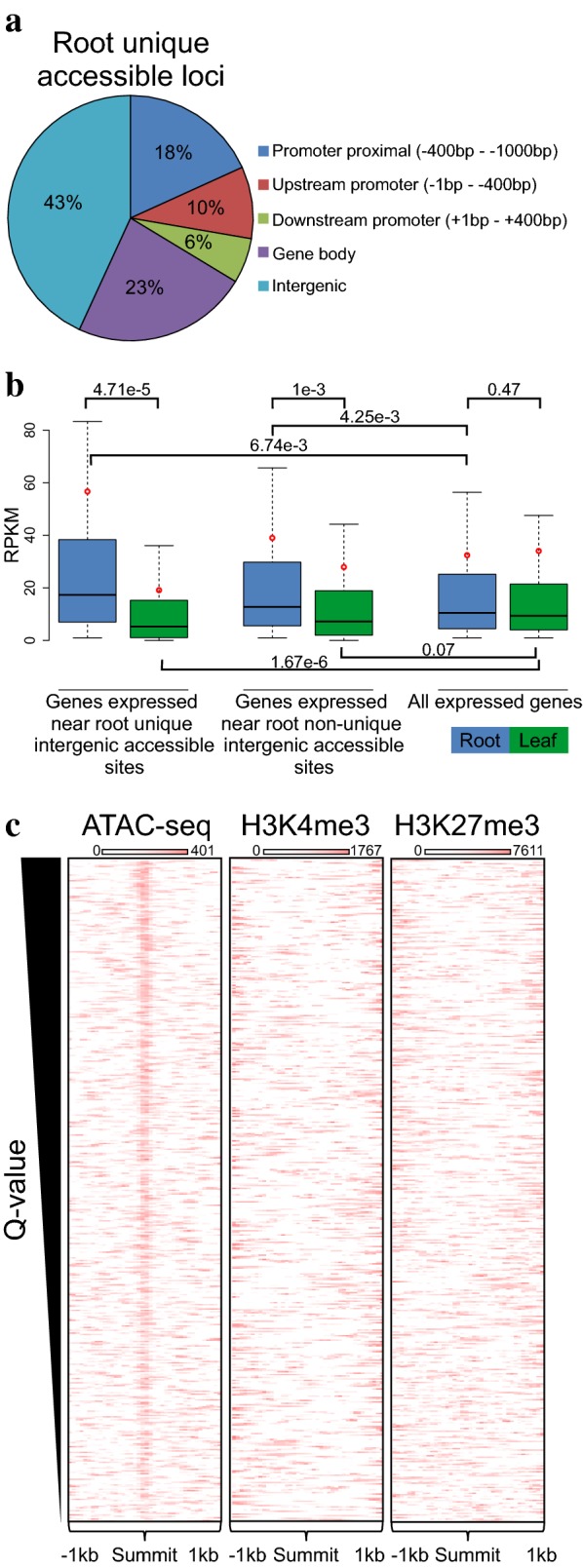
Intergenic accessible loci are linked to expression of distant genes. **a** Genic distribution of root-unique accessible sites. **b** Boxplots of expression level (only for genes with RPKM > 1) of genes near root unique intergenic accessible sites, root nonunique intergenic accessible sites and all expressed genes in roots (blue) and leaves (green). The horizontal line represents the median, and the red circle represents the mean of each dataset. Student’s t-test p-value is presented on the bars connecting each pair tested. **c** Heatmaps of ATAC-seq, H3K4me3 ChIP-seq, and H3K27me3 ChIP-seq signals at root intergenic accessible loci. Loci were ranked according to the ATAC-seq peak Q-value and centered at the summits of ATAC-seq peaks

Given that root-unique accessible sites seem to contribute to root-specific transcription factor binding, we sought to analyze them to further understand how they are related to gene transcription in roots. Interestingly, root-unique loci are predominantly intergenic (43%). This proportion of intergenic loci is doubled relative to the complete dataset of accessible chromatin (23%) (Fig. 6a), which is noteworthy given the high gene density of the *Arabidopsis* genome. To test the linkage of intergenic loci to genes we hypothesized that they may represent enhancers of distant genes, we made the naïve assumption that each accessible site regulates the expression of the nearest gene. The vast majority of the genes in proximity to root-unique or nonunique accessible sites are expressed (RPKM > 1, 79% of 1039, p < 2.2e−16 and 72% of 5551, p < 2.2e−16, respectively. Fisher’s exact test was used, given that 56% of genes are expressed genome wide). In addition, the average expression level of genes near root unique or nonunique intergenic accessible sites (56 and 39 RPKM, respectively) is significantly increased compared with the genome-wide average (32 RPKM, p-value = 6.74e−3 and p-value = 4.25e−3, respectively, Student’s t-test), suggesting that intergenic chromatin accessibility is linked to gene expression in roots (Fig. 6b). Given that genes associated with both groups of intergenic sites are highly expressed in roots, we conclude that these sites are likely regulatory sites of distant genes in roots. This notion is supported by the fact that the organ specificity in chromatin accessibility is mirrored in organ-specific gene expression. The average expression of genes near root unique intergenic accessible sites in leaves is reduced compared with that in roots (average 56 RPKM in roots versus 19 RPKM in leaves, p-value = 4.7e−5, Student’s t-test) and the genome-wide average in leaves (19 and 34 RPKM, respectively, p-value = 1.67e−6, Student’s t-test). This magnitude of difference (p-value = 1.043e−15, Student’s t-test) in the expression level is not found for the genes near nonunique intergenic sites (mean of 39 RPKM in roots versus 28 RPKM mean in leaves, p-value < 0.001, Student’s t-test) or genome-wide expression (32 RPKM mean in roots and 34 RPKM mean leaves, p-value = 0.47, Student’s t-test) (Fig. 6b), suggesting that the root-unique intergenic accessible sites contribute to root-unique gene expression.

Consistent with their expected role as putative regulatory elements, intergenic loci are not associated with H3K4me3 or H3K27me3 gene histone marks (Fig. 6c). To further investigate whether these intergenic loci contain features that are related to transcription factor binding, we performed motif discovery analysis. Similar to the entire root-unique loci that contain also TSS-proximal loci, we identified the motif of the MYB61 TF (rank 4, p-value = 1e−4), indicating the relevance of this approach. Notably, the list from this focused approach also includes candidate TFs with an as yet unknown role in gene regulation in roots. For example, MYB49 is expressed in roots (tenfold increase compared with leaves) and was up regulated in response to salt stress [46]. Although the MYB49 motif was uncovered in both enrichment lists, it is ranked 195 (p-value = 1e−30) in the global root-unique loci and 9 (p-value = 1e−4) in the list of root-unique intergenic loci (Additional file 1: Table S3).

Altogether, focusing on organ-specific gene-distant sites may reveal novel transcription factors that are important for defining root function or identity. In addition, the fact that the potential binding sites of these factors are distant from genes suggests that they may reach their gene targets by chromosome looping [47].

## Discussion

It has been long known that the genomic potential relies not only on the DNA sequence but also on epigenetic features, such as chromatin accessibility to TF binding. Therefore, the chromatin accessibility landscape of the *Arabidopsis thaliana* genome reveals valuable information about active CREs and the TFs that bind them and further enables study of the relationship between CREs and gene expression.

ATAC-seq is a novel technology that can be applied using a small quantity of nuclei [12], thereby overcoming the technological hindrance of other powerful technologies, such as DNaseI-seq, in plant genomic research. Adapting ATAC-seq to plants requires methods to avoid DNA degradation by nucleases released from the vacuole. This is achieved by coupling ATAC-seq with INTACT that allows rapid isolation of nuclei with the benefit of low plastid and mitochondrial DNA contamination. We obtained a remarkably low contamination (~ 1.76% on average) compared with 30–70% in animal cells and 22.3% in fluorescence-sorted *Arabidopsis* nuclei [22]. Coupling INTACT and ATAC-seq in a single pipeline enables profiling of cell-type-specific chromatin accessibility. Given the wide implementation of the INTACT method in plant research, tools for characterizing tens of different cell types from various plant species are readily available [24, 48–50].

In addition, we present a rapid qPCR assay for assessing the quality of ATAC-seq libraries prior to high-throughput sequencing. This assay conserves resources and is particularly valuable when calibrating ATAC-seq for novel organisms or cell types [37]. Notably, the sites we designed to use as positive or negative controls based on available data were found to be accessible in more recent ATAC-seq experiments in various tissues [16, 22, 25]. These findings indicate the robustness of the qPCR-based approach.

High chromatin accessibility was observed at promoters of expressed genes, which are also decorated with the H3K4me3 histone mark but not with the H3K27me3 mark, which is similar to chromatin accessibility in rice [51].

Identifying sequence motifs enriched in chromatin accessible loci allows identification of active transcription factors and their postulated binding sites, and focusing on organ-specific regulatory chromatin increases the discovery power of organ-specific TFs. Thus, dissecting an organ to specific cell-types is expected to increase our understanding of cell-type specific regulatory DNA elements and their associated transcription factors. This approach for identifying potentially relevant transcription factors is unbiased and global. However, this method needs to be complemented by more direct methods, such as ChIP-seq for measuring transcription factor binding, given that binding at some loci may be indirect. In addition, the motif signature is not similarly abundant for different TFs, and binding at some loci may be indirect [52].

Approximately one-fourth of the accessible sites are located within 400 bp upstream of a TSS, where the basal transcription machinery binds. Other accessible sites are distributed downstream and upstream to the TSS within gene bodies and at intergenic regions. The proportional distribution of accessible sites relative to the TSS is similar in *Arabidopsis* roots and leaves. However, the proportion of accessible sites at the TSS was reduced by greater than twofold in the root-unique sites. This finding may reflect the fact that promoter proximal accessible sites are correlated with transcriptional activity and that the majority of expressed genes (~ 90%) are shared between the two organs. The high organ-specific expression of transcription factors [53] may suggest that similar genes are regulated by different TFs in the two organs. This notion is supported by the fact that the group of TSS-distant regulatory elements exhibits the highest organ-specificity relative to all other genic loci. Notably, despite their intergenic position, root unique loci tend to be located near genes with a higher expression in roots relative to leaves, suggesting their role as distant transcriptional regulators. Indeed, this focused group of accessible loci are enriched with binding motifs for known root TFs as well as currently unknown TFs. Finally, the finding that these regulatory sites are distant from genes despite the high gene density in *Arabidopsis thaliana* supports the hypothesis that these regulatory sites communicate with their gene targets by chromosome looping, a concept that is emerging in plant research [54, 55].

## Conclusions

We present a robust pipeline to profile accessible chromatin in *Arabidopsis* by combining INTACT with ATAC-seq. This methodology can be rapidly applied to multiple cell types, plant species and conditions. The resulting chromatin accessibility profile covers various genomic activities. TSS-proximal loci are associated with gene expression levels and histone marks of gene activity. Intergenic accessible loci are primarily enriched with TF binding and TF binding motif sequences. TSS-distant regulatory elements exhibited the highest organ specificity compared with TSS-proximal loci or gene expression. Furthermore, these organ-specific accessible loci are linked to distant genes with organ-enhanced expression, suggesting that they are functional organ-specific cis regulatory elements. Altogether, this finding suggests that organs or cell types are shaped primarily by variation in regulatory chromatin relative to variation in gene expression profiles.

## Additional file

**Additional file 1: Fig. S1.** Browser view of a representative locus chosen as a positive QC. **Table S1.** Mapping and peak calling results for the three biological repeats shown in Fig. 2. **Table S2.** GO analysis of biological processes of highly expressed genes near root-unique accessible sites. **Table S2.** Top 30 sequence motifs enriched in the root ATAC-seq peaks. **Table S3.** TF binding motifs enriched in root-unique ATAC-seq peaks.

## Abbreviations

ATAC-seq: assay for transposase-accessible chromatin with high throughput sequencing
BCCD: biotin carboxyl carrier protein domain
ChIP-seq: chromatin immunoprecipitation sequencing
CRE: *cis*-regulatory elements
DHS: DNaseI hypersensitivity site
FC: fold change
GO: gene ontology
HPB: HA tag–PreScission–Biotin
INTACT: isolation of nuclei tagged in specific cell types
MS: Murashige and Skoog
NGS: next-generation sequencing
NPB: nuclear purification buffer
TF: transcription factor
TSS: transcription start site
