# Supplementary material for: Regulatory chromatin landscape in Arabidopsis thaliana roots uncovered by coupling INTACT and ATAC-seq
Source: Plant Methods. 2018 Dec 20;14:113. doi: 10.1186/s13007-018-0381-9 (PMC6300899; doi:10.1186/s13007-018-0381-9)
Supplement: Supplementary file 1 — Additional file 1: Fig. S1. Browser view of a representative locus chosen as a positive QC. Table S1. Mapping and peak calling results for the three biological repeats shown in Fig. 2. Table S2. GO analysis of biological processes of highly expressed genes near root-unique accessible sites. Table S2. Top 30 sequence motifs enriched in the root ATAC-seq peaks. Table S3. TF binding motifs enriched in root-unique ATAC-seq peaks. [file 13007_2018_381_MOESM1_ESM.pptx]

## Slide 1
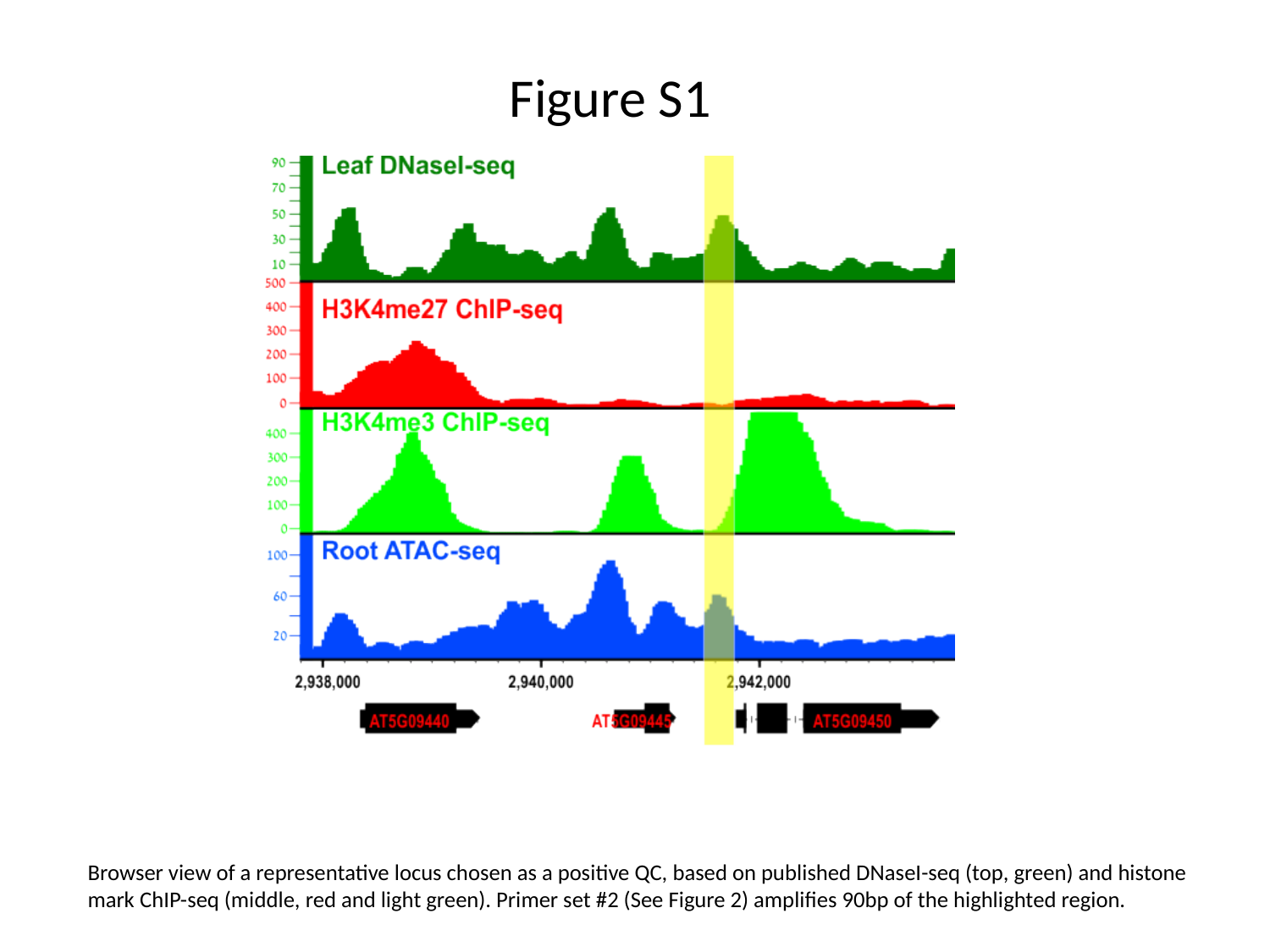

Figure S1
Browser view of a representative locus chosen as a positive QC, based on published DNaseI-seq (top, green) and histone mark ChIP-seq (middle, red and light green). Primer set #2 (See Figure 2) amplifies 90bp of the highlighted region.

## Slide 2
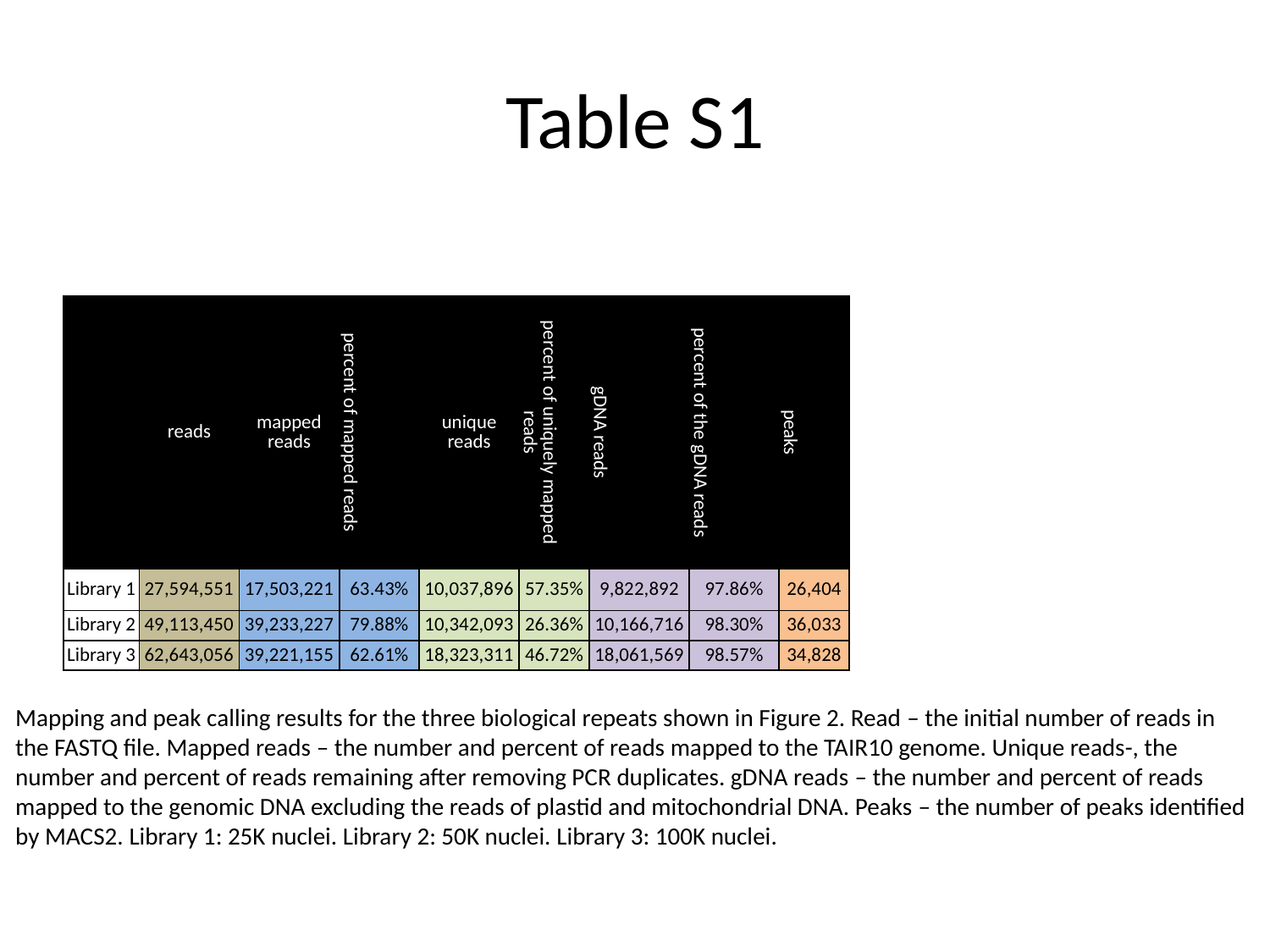

# Table S1
| | reads | mapped reads | percent of mapped reads | unique reads | percent of uniquely mapped reads | gDNA reads | percent of the gDNA reads | peaks |
| --- | --- | --- | --- | --- | --- | --- | --- | --- |
| Library 1 | 27,594,551 | 17,503,221 | 63.43% | 10,037,896 | 57.35% | 9,822,892 | 97.86% | 26,404 |
| Library 2 | 49,113,450 | 39,233,227 | 79.88% | 10,342,093 | 26.36% | 10,166,716 | 98.30% | 36,033 |
| Library 3 | 62,643,056 | 39,221,155 | 62.61% | 18,323,311 | 46.72% | 18,061,569 | 98.57% | 34,828 |
Mapping and peak calling results for the three biological repeats shown in Figure 2. Read – the initial number of reads in the FASTQ file. Mapped reads – the number and percent of reads mapped to the TAIR10 genome. Unique reads-, the number and percent of reads remaining after removing PCR duplicates. gDNA reads – the number and percent of reads mapped to the genomic DNA excluding the reads of plastid and mitochondrial DNA. Peaks – the number of peaks identified by MACS2. Library 1: 25K nuclei. Library 2: 50K nuclei. Library 3: 100K nuclei.

## Slide 3
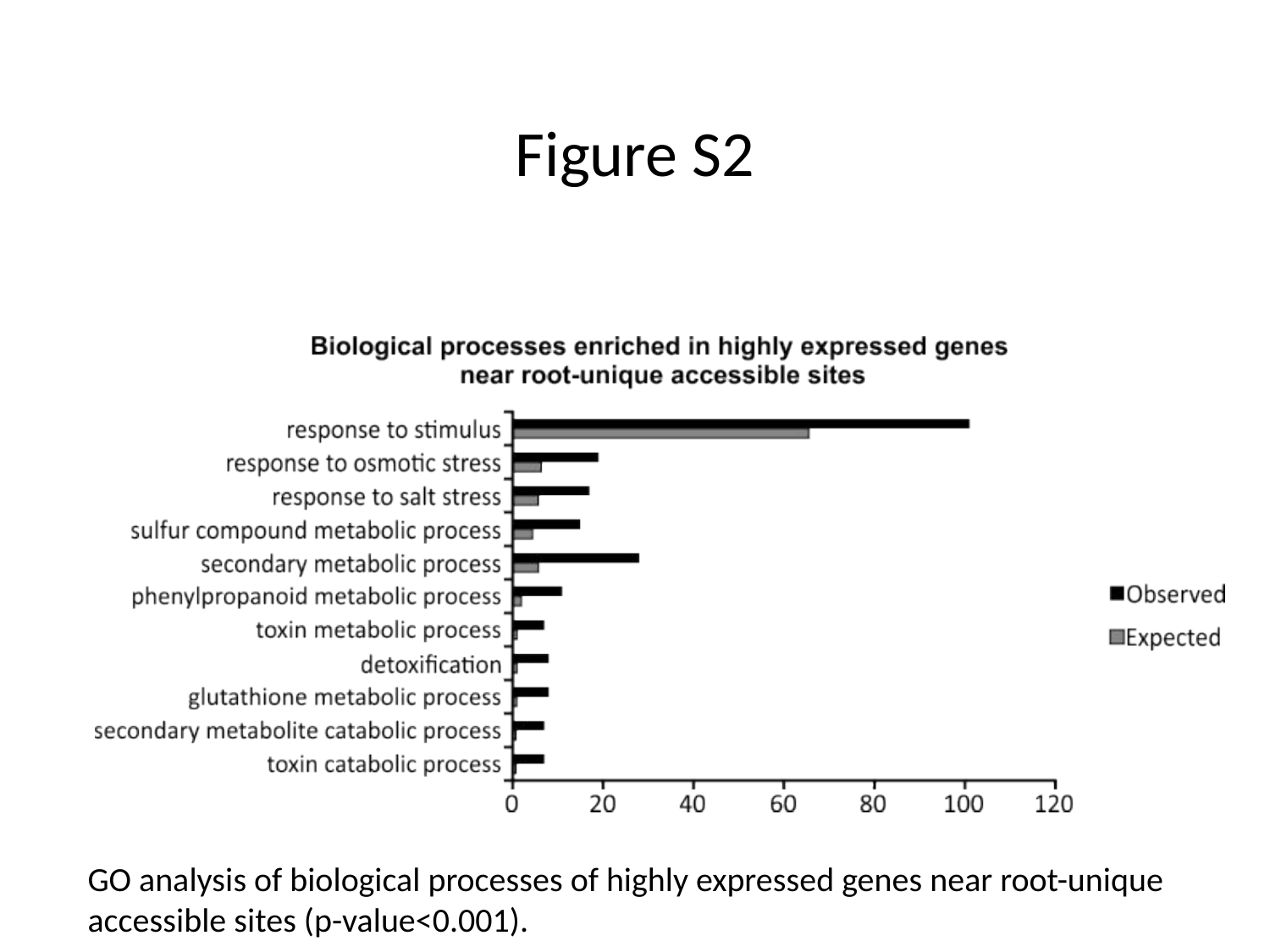

# Figure S2
GO analysis of biological processes of highly expressed genes near root-unique accessible sites (p-value<0.001).

## Slide 4
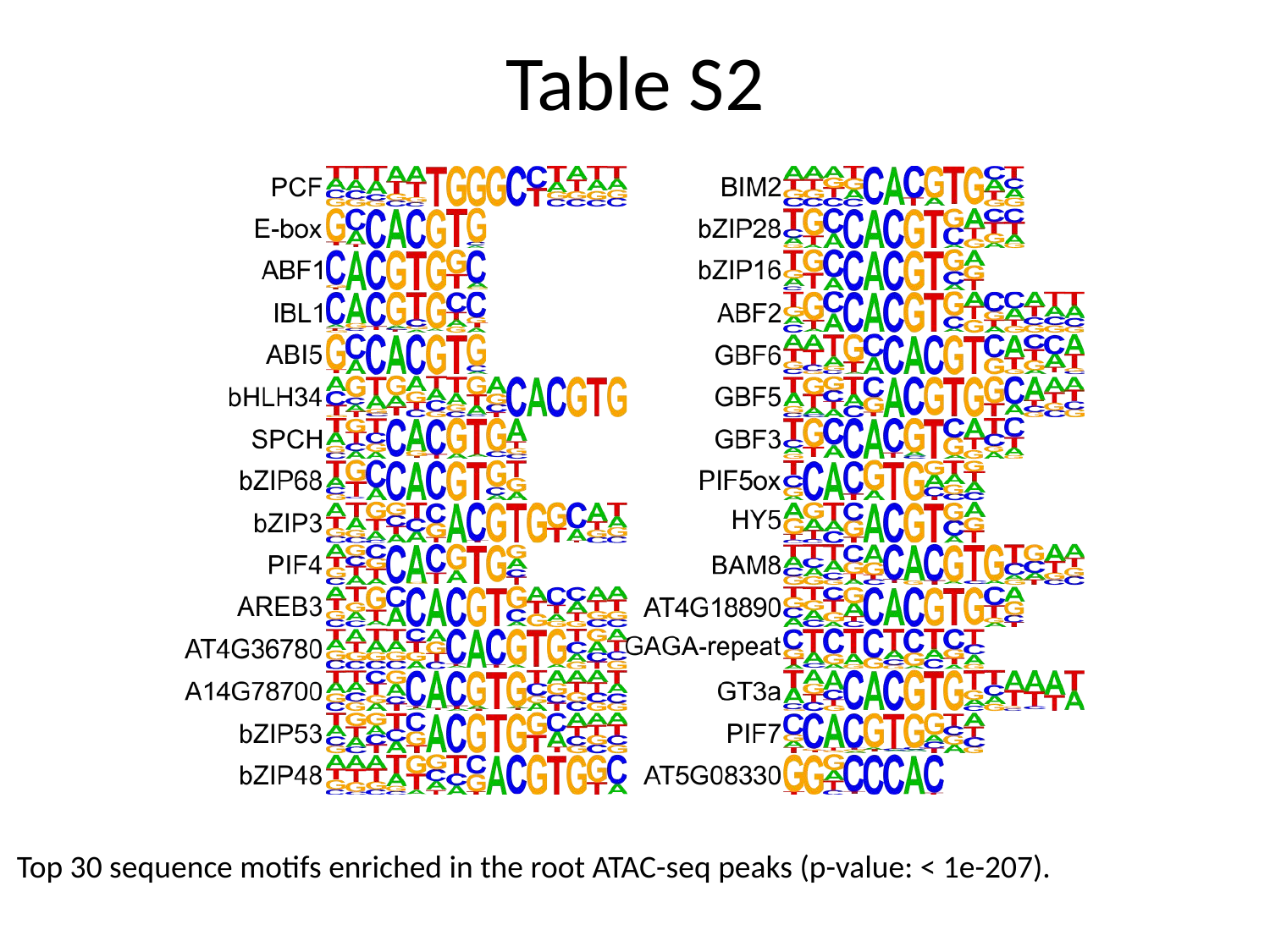

# Table S2
Top 30 sequence motifs enriched in the root ATAC-seq peaks (p-value: < 1e-207).

## Slide 5
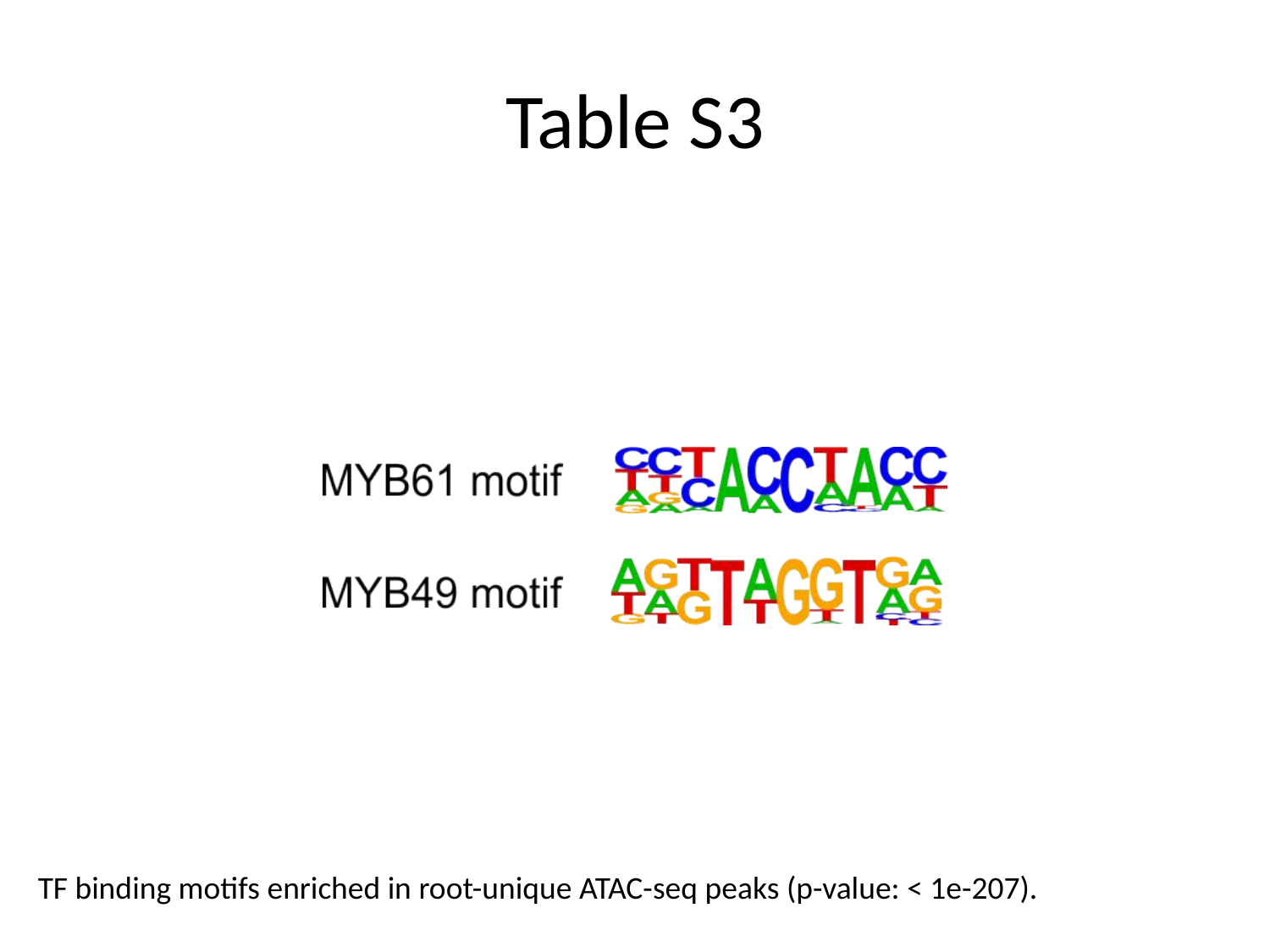

# Table S3
TF binding motifs enriched in root-unique ATAC-seq peaks (p-value: < 1e-207).
